# Elevated Oxytocin Receptor Blood Concentrations Predict Higher Risk for, More, and Earlier 24-Month Hospital Readmissions after In-Patient Detoxification in Males with Alcohol Use Disorder

**DOI:** 10.3390/ijms23179940

**Published:** 2022-09-01

**Authors:** Christiane Mühle, Massimiliano Mazza, Christian Weinland, Claudia von Zimmermann, Patrick Bach, Falk Kiefer, Valery Grinevich, Iulia Zoicas, Johannes Kornhuber, Bernd Lenz

**Affiliations:** 1 Department of Psychiatry and Psychotherapy, Friedrich-Alexander University Erlangen-Nürnberg (FAU), Schwabachanlage 6, D-91054 Erlangen, Germany; 2 Department of Addictive Behavior and Addiction Medicine, Central Institute of Mental Health (CIMH), Medical Faculty Mannheim, Heidelberg University, J 5, D-68159 Mannheim, Germany; 3 Department of Neuropeptide Research in Psychiatry, Central Institute of Mental Health (CIMH), Medical Faculty Mannheim, Heidelberg University, J 5, D-68159 Mannheim, Germany

**Keywords:** oxytocin receptor, alcohol relapse, alcohol use disorder, alcohol dependence

## Abstract

Alcohol use disorder (AUD) is a major global mental health challenge. Knowledge concerning mechanisms underlying AUD and predictive biomarkers of AUD progression and relapse are insufficient. Recently, addiction research is focusing attention on the oxytocin system. However, to our knowledge, blood concentrations of the oxytocin receptor (OXTR) have not yet been studied in AUD. Here, in sex-separated analyses, OXTR serum concentrations were compared between early-abstinent in-patients with AUD (113 men, 87 women) and age-matched healthy controls (133 men, 107 women). The OXTR concentrations were correlated with sex hormone and oxytocin concentrations and alcohol-related hospital readmissions during a 24-month follow-up. In male patients with AUD, higher OXTR concentrations were found in those with an alcohol-related readmission than in those without (143%; *p* = 0.004), and they correlated with more prospective readmissions (ρ = 0.249; *p* = 0.008) and fewer days to the first readmission (ρ = −0.268; *p* = 0.004). In men and women, OXTR concentrations did not significantly differ between patients with AUD and controls. We found lower OXTR concentrations in smokers versus non-smokers in female patients (61%; *p* = 0.001) and controls (51%; *p* = 0.003). In controls, OXTR concentrations correlated with dihydrotestosterone (men, ρ = 0.189; *p* = 0.030) and testosterone concentrations (women, ρ = 0.281; *p* = 0.003). This clinical study provides novel insight into the role of serum OXTR levels in AUD. Future studies are encouraged to add to the available knowledge and investigate clinical implications of OXTR blood concentrations.

## 1. Introduction

Alcohol use disorder (AUD) is among the most prevalent global mental health challenges [1]. To date, we lack sufficient understanding of the mechanistic underpinnings of AUD and the role of predictive biomarkers of AUD progression.

Recently, the scientific community has increased its focus on the oxytocin (OXT) system, which is composed of a central and peripheral distribution of the nonapeptide OXT and the OXT receptor (OXTR). The OXT system mediates a vast number of both animal and human behavioral and physiological effects, including regulation of socio-sexual and socio-emotional behavior in addition to hedonistic feeding and drug-seeking behaviors [2]. The OXTR is expressed in multiple brain regions. High levels of *OXTR* mRNA expression were observed in the amygdala [3], an area of the brain that has a critical role in mediating anxiety and stress-responsiveness. OXT and the OXTR are involved in regulation of anxiety, stress, and reward-related behaviors [2,4,5].

Addiction research is focusing its attention on the role of the OXT system since studies have shown its involvement in mechanisms relevant to the development and maintenance of addiction. These addiction-related systems include dopamine signaling, activation of gamma-aminobutyric acid (GABA)-ergic interneurons, glutamate signaling in the brain, and activation of the hypothalamic–pituitary–adrenal (HPA) [6,7] and hypothalamic–pituitary–gonadal (HPG) axes [8]. Via reduction of increased symptoms of stress, anxiety, and social isolation commonly associated with alcohol withdrawal [9,10,11], increased OXT system activity could potentially lead to a reduction in the severity of withdrawal symptoms [12,13] and thereby aid in the prevention of alcohol addiction relapse.

The OXTR has been localized to the mesolimbic dopamine system, which is an essential mediator of the reward neurocircuitry, involved in promoting the development of addiction [2,7]. Rodent models suggest that intracerebroventricular administration of OXT reduces alcohol consumption in addition to alcohol-induced dopamine efflux in the nucleus accumbens (NAc) [14,15]. The OXTR is found in the NAc, ventral tegmental area (VTA) projecting to the NAc [16], medial prefrontal cortex (mPFC), amygdala, and hippocampus. Additionally, OXTR is present on dopamine neurons that project from the VTA to mPFC [16,17]. Interestingly, recent investigations have found evidence of an OXTR/dopamine 2 receptor complex (OXTR-D2R) within the NAc. In this area, OXT acts as an allosteric agonist that leads to an increase in D2R affinity. Activation of D2R reduces drug-seeking behavior, and DR2 undergoes significant downregulation following chronic drug exposure [5]. Hence, the OXTR–D2R interaction after its activation by OXT may cause a reduction in drug-seeking behavior [18].

The OXT system also influences GABAergic interneurons. They express excitatory Gq-coupled OXTR in the NAc, hippocampus, and PFC. Hence, OXT may influence drug seeking behavior and dopaminergic signaling through these GABAergic interneurons by increasing the inhibitory tone within these brain areas [5]. In addition, it is known that OXTR is located on GABAergic interneurons in the NAc [19], which in turn regulate drug-seeking behaviors [20]. Hence, it may be posited that OXT impacts drug-seeking behavior directly through interactions with the OXTR on GABAergic interneurons. This process occurs in areas that are critical to dopamine signaling and addiction processes [5].

OXT signaling is also relevant to glutamatergic neurons projecting from the PFC to the NAc and VTA, which are thought to regulate cue-induced reinstatement of drug-seeking behaviors [21]. OXT causes a decrease in methamphetamine-induced glutamate release by binding to the OXTR [22]. The OXT–OXTR interaction is thought to attenuate changes in glutamatergic neurotransmission partially via regulation of glutamatergic receptors in the PFC after acute methamphetamine administration in mice. Moreover, the administration of the selective OXT inhibitor atosiban entails opposite effects by antagonizing the effects of OXT [22]. The importance of the glutamatergic system in the treatment of AUD is highlighted by the application of acamprosate, a glutamate modulator that is used in clinical practice to prevent alcohol-related relapses [23].

Finally, interactions between the OXT system and the HPA [6] and HPG [8] axes are established. Concurrent stress-induced release of OXT within the paraventricular nucleus of the hypothalamus and plasma corticosterone was measured [24], and OXT is believed to exert an inhibitory function on the hypothalamic expression of corticotropin releasing factor as well as on HPA axis activation [25]. Moreover, the OXT system interfaces with the HPG axis by interacting with sex hormones and their respective receptors [8], which in turn are involved in AUD [26,27,28,29,30,31,32]. For example, the administration of androgens and estrogens to castrated rats increases *OXTR* mRNA in the ventromedial hypothalamus [33,34]. In an OXTR-dependent manner, allopregnanolone, the primary metabolite of progesterone, and to a lesser extent also 17-beta-estradiol, evoke a robust Ca^2+^ influx in postnatal but not adult supraoptic nucleus neurons leading to the release of OXT. This effect is opposite in the peripheral nervous system in which allopregnanolone and progesterone inhibit the GABA-induced Ca^2+^ increase in embryonic dorsal root ganglion neurons [35].

Thus, OXT and OXTR blood concentrations are of great interest to human research. Blood concentrations can be easily measured. Central and peripheral OXT concentrations correlate with each other after intranasal OXT administration and in stressful situations [36]. In addition to its expression in brain regions, the OXTR has also been found in numerous peripheral cells and tissues, including adrenal medulla cells [37], macula densa cells of the renal cortex [38], cardiomyocytes [39], osteoclasts and osteoblasts [40], adipocytes [41], and the myometrium [42]. Moreover, the OXTR was quantified in the blood, especially in peripheral blood mononuclear cells, such as lymphocytes and macrophages at mRNA [43,44,45] and protein [44,45] levels. To our knowledge, no published data on serum or plasma OXTR protein concentrations in humans or animals except for pregnant women [46] are available and the relationship between central and peripheral OXTR levels in humans is unknown. An indication of a differential regulation is provided from male mice exposed to 6 h cold stress with an increased expression in the brain versus a decrease in testis [47].

A strong need for biomarkers to predict the outcome of patients with AUD following alcohol withdrawal exists, and a line of studies suggests that the OXT system might provide such predictors. Hansson et al. [48] found evidence that intracerebroventricular administration of OXT reduces cue-induced alcohol relapse-like behavior in alcohol-dependent male rats but not in female rats [49]. In a randomized cross-over trial, the administration of intranasal OXT caused a decrease in the connectivity of the NAc in an fMRI alcohol cue–exposure paradigm [50], which is important as higher striatal cue-exposure has been established to predict relapse [51]. A recent human study observed higher OXT concentrations as a predictor of more readmissions and fewer days to the first readmission during a 24-month period in male patients with AUD [52]. Notwithstanding, we lack important information about the potential of the easily accessible OXTR blood concentrations to predict the outcomes following withdrawal treatment in AUD patients. Such knowledge would inform the establishment of future prevention and treatment strategies.

### Aims of the Study

To summarize, the literature indicates that the OXT system influences neurobiological mechanisms of addictive behaviors and may also serve as a predictor of relapse in patients with AUD. As far as we know, this study is the first one to investigate cross-sectional and longitudinal differences in OXTR serum concentrations in in-patients with AUD and healthy control subjects, along with use of OXTR concentrations to predict alcohol-related readmissions. As the HPG axis interacts with the OXT system, and data on androgens, progesterone, and estrogens were available in this cohort [29,31,32], we also explored associations between the measured OXTR concentrations and sex hormone concentrations. Research on AUD in women has been neglected so far. Hence, we actively recruited a sex-balanced cohort and conducted sex-specific analyses to provide the urgently needed evidence separated for men and women.

## 2. Results

### 2.1. Demographic Characteristics

The male and female groups of in-patients with AUD did not significantly differ from the sex-specific healthy control group in terms of age, fasting status, and postmenopausal status. As expected, relative to sex-specific healthy controls, the male and female patients showed higher carbohydrate-deficient transferrin (CDT) levels (men, 194%; women, 132%) and were more likely smokers (odds ratio [OR] for men, 12.6; for women, 14.5; Table 1).

### 2.2. Oxytocin Receptor Blood Concentrations

In both men and women, the OXTR concentrations did not significantly differ between patients with AUD and healthy control subjects, and the OXTR concentrations also did not significantly change during early withdrawal (from baseline to follow-up, Table 2).

**Figure 1:**
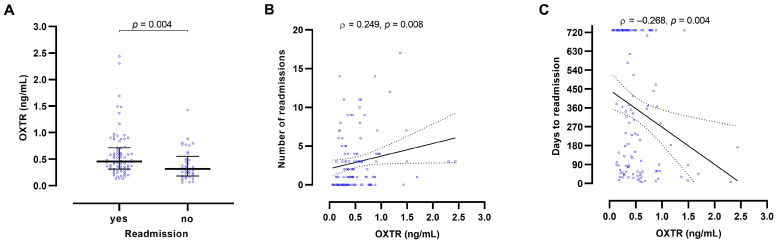
Oxytocin receptor (OXTR) blood concentrations predict 24-month alcohol-related hospital readmissions in male in-patients with alcohol use disorder. We found higher baseline OXTR concentrations in in-patients with a prospective alcohol-related hospital readmission than in those without readmission (**A**). Higher baseline OXTR concentrations predicted more alcohol-related readmissions during the 24-month period (**B**) and fewer days to first alcohol-related readmission (**C**). The graphs present medians with interquartile range and *p* value from a Mann–Whitney U test (**A**) and ρ and *p* values from Spearman correlations and best-fit lines from regression analysis with 95% confidence intervals (**B**,**C**).

We found higher (143%) baseline OXTR concentrations in male patients with any alcohol-related readmission during the 24-month follow-up than in male patients without any readmission (*p* = 0.004). Assigning individuals with OXTR concentrations equal or above the Youden cut-off point of 0.351 ng/mL to the group with readmission resulted in a sensitivity of 0.71 and a specificity of 0.59 (area under the curve 0.666, standard error under the non-parametric assumption 0.056, *p* = 0.003). In male patients, higher baseline OXTR concentrations also correlated with more prospective alcohol-related readmissions (ρ = 0.249; *p* = 0.008) and fewer days to the first readmission (ρ = −0.268; *p* = 0.004) (Figure 1). However, OXTR levels were not significantly associated with markers of alcohol dependence history or severity (alcohol concentration at admission, number of previous withdrawal treatments, lifetime and daily ethanol consumption, and liver parameters (glutamic-oxaloacetic transaminase (GOT), glutamic-pyruvic transaminase (GPT), gamma-glutamyl transferase (GGT) activities; data not shown) and had thus a predictive value independent from easily accessible markers in clinical practice.

To assess the predictive potential of both parameters, receptor and ligand of the OXT system, we subdivided the male patients depending on the male-specific Youden cut-off points into low- and high-risk groups of alcohol-related hospital readmission during the 24-month follow-up period. Whereas in the group of the male patients with low OXTR serum concentrations, 51.4% of those with low OXT concentrations and 42.9% with high OXT concentrations were readmitted, the rates increased to 69.4% and 87.9% for high OXTR combined with low and high OXT levels, respectively (Table S1). These data support a significant interaction of both parameters and could suggest a functional role of serum OXTR with a complex adaptation within the system.

In female patients, baseline OXTR concentrations were not significantly associated with alcohol-related readmission during the 24-month follow-up (Tables S2 and S3).

Smoking behavior was not found to be significantly associated with OXTR concentrations in male patients or male control subjects. However, OXTR concentrations were lower in smoking versus non-smoking female patients (61%; *p* = 0.001) and control groups (51%; *p* = 0.003) (Table S4), and the levels were also negatively associated with Fagerström Test for Nicotine Dependence (FTND [53,54]) scores in female patients (N = 75; ρ = −0.414; *p* < 0.001). In accordance with the change in OXTR binding densities in specific brain regions [55] with growing age, we found that lower baseline OXTR concentrations correlated with higher age in healthy control subjects (men, ρ = −0.192; *p* = 0.027; women, ρ = −0.246; *p* = 0.011; Table S5).

In terms of sex hormones, higher OXTR concentrations correlated with higher dihydrotestosterone concentrations in male controls (ρ = 0.189; *p* = 0.030) and with higher testosterone concentrations in female controls (ρ = 0.281; *p* = 0.003). Beyond those findings, no significant correlations between OXTR concentrations and dihydrotestosterone, testosterone, estradiol, or progesterone concentrations were found (Table S6). Blood OXTR concentrations also did not significantly differ between men and women either in patients (baseline or follow-up) or in controls (data not shown).

Finally, we found a significant correlation between baseline OXTR concentrations and baseline OXT concentrations in the male patient group (N = 113; ρ = 0.331; *p* < 0.001), but not in the other groups (female patients: N = 87; ρ = 0.008; *p* = 0.942; male controls: N = 133; ρ = −0.052; *p* = 0.554; female controls: N = 107; ρ = 0.065; *p* = 0.505).

## 3. Discussion

A growing body of evidence highlights a role of the OXT system in AUD. However, to our knowledge, this study is the first one to systematically assess OXTR blood concentration as a peripheral marker for the activity of the OXT system in patients with AUD compared to healthy controls. We used a unique cross-sectional, longitudinal, and sex-separated study design. To counteract the significant underrepresentation of women in AUD research, in this study we actively enrolled a relatively large sample of women with AUD in order to provide separate evidence for men and women. Particularly in the context of AUD and the OXT system, large sex-dependent effects have been reported. These differences highlight the importance of sex-separated investigations and, for example, limit the performance of randomized clinical trials with OXT-based interventions in male AUD patients only [49].

AUD often runs a chronic course with frequent relapses and alcohol-related hospital readmissions. This study established higher baseline OXTR concentrations in male patients with at least one alcohol-related hospital readmission during the 24-month follow-up than in patients without any readmission (143%). In male patients, higher baseline OXTR blood concentrations were also found to correlate with a higher number of readmissions during the follow-up and fewer days to the first readmission. Notably, OXTR concentrations appear to provide an independent predictive value and were not a surrogate marker of routinely collected anamnestic or clinical laboratory parameters of alcohol consumption. In contrast, in female patients, baseline OXTR concentrations were not significantly associated with any parameters of alcohol-related readmission. Our previous work on AUD shows an association of higher baseline OXT concentrations with more readmissions during the 24-month period and fewer days to the first readmission also in male patients [52]. These results are strengthened by the combined predictive effect of OXT and OXTR levels for the readmission rate in the present study. The data indicate that in male patients with AUD, a higher activity of the OXT system may serve as a predictor of alcohol-related hospital readmission. The sex-specific effects observed here also highlight the importance of conducting AUD research separately for men and women.

While we have previously detected elevated levels of OXT in both male and female patients with AUD, which normalized during early withdrawal [52], we did not observe a significant difference in OXTR concentrations between male and female AUD in-patients and healthy controls. In addition, OXTR concentrations did not significantly change during early withdrawal, namely from baseline to the at median 5-day follow-up. However, a power analysis indicated that our cohort was sufficiently large to detect differences with small to medium effect sizes of Cohen’s d values of 0.36 and 0.41 (G*Power; 2-tailed, α error probability 0.05, power 0.80) for men and women, respectively. On the one hand, OXTR binding sites for radiolabeled ornithine vasotocin analog were markedly upregulated in brain tissues of deceased male alcohol-dependent patients (ventral striatum and nucleus caudatus) and male rats (caudate putamen) most likely caused by the reduced OXT expression (detected by immunoreactivity) in hypothalamic paraventricular and supraoptic nuclei [48]. These changes were not present in female alcohol-dependent patients and rats [49]. On the other hand, a similar lack of group differences between subjects with AUD and controls as in our study was found for *OXTR* mRNA expression levels in all five analyzed brain regions of post-mortem samples (NAc, VTA, PFC, amygdala, and hippocampus), whereas *OXT* mRNA was significantly higher only in the PFC of AUD patients compared to controls and correlated positively with daily alcohol intake and drinks per week [56]. This finding might suggest that in contrast to the OXT hormone levels, the blood concentrations of the corresponding receptor do not respond to chronic alcohol consumption or withdrawal treatment, possibly leading to a shift in the balance of ligand and receptor. Underlying mechanisms might involve desensitization of the OXTR. However, it might also be that long-term abstinent patients with AUD differ from healthy control subjects and that these differences disappear during chronic alcohol consumption. Moreover, brain and blood OXTR concentrations could be differentially regulated as observed at the mRNA level in mice exposed to cold stress [47], and they may follow different time courses, possibly also lagging behind OXT levels. Future research is needed to clarify this issue.

Furthermore, we assessed whether smoking status is associated with OXTR concentrations. We found lower OXTR concentrations in female AUD patients (61%) and control group (51%) smokers as opposed to non-smokers. In the female patients, lower OXTR concentrations were also associated with higher severity of smoking as indicated by FTND scores. The analyses were conducted separately in the patient and control groups to show that the observed effects of smoking are independent from alcohol use. The observed association between lower OXTR concentrations and smoking in women are supported by a recent finding of lower OXT concentrations in smoking in comparison to non-smoking women [52]. From a mechanistic point of view, smoking-induced alterations in HPA axis activity might account for this association. Elevated cortisol levels in smokers versus non-smokers are well-established [57] and preclinical research demonstrated that environmental stressors downregulate *OXTR* expression in both peripheral and brain tissues of zebrafish [58]. On the other hand, Kanamori et al. [59] reported increased OXTR concentrations in the uterine myometrium in smoking versus non-smoking pregnant women and positive correlations with the number of daily cigarettes consumed and the concentration of exhaled carbon monoxide. This observation may seem initially contradictory. However, these findings do not necessarily indicate a theoretical inconsistency as they may highlight the diverse regulation and function of the *OXTR* expression in various peripheral tissues. Certainly, future research is needed to enlighten the mechanisms underlying the observed lower OXTR blood concentrations in smoking versus non-smoking women. In our male AUD and control groups, smoking behavior was not significantly associated with OXTR concentrations.

For validation purposes, we analyzed the effect of age and found that lower OXTR blood concentrations correlated with higher age in healthy control subjects. These findings are in line with changes in OXTR binding densities observed in specific brain regions with growing age [55]. The fact that OXTR concentrations did not correlate with higher age in patients with AUD might be due to an interfering effect caused by AUD.

Due to the known interactions of the OXT system with steroid hormones [8,35], we were interested in associations between OXTR concentrations and sex hormones. We observed positive correlations with dihydrotestosterone in male controls and with testosterone in female controls. Our data did not yield a significant association of estradiol or progesterone with OXTR concentrations, suggesting a more crucial role of the androgen system in AUD. The sex-specific dihydrotestosterone and testosterone findings could be explained by the fact that dihydrotestosterone is produced in greater abundance in men, while in the female population a greater quantity of dihydrotestosterone is found in its inactive form, bound to sex hormone-binding globulin [60]. Our clinical observation of higher OXTR concentrations in subjects with higher androgen concentrations is supported by a preclinical study demonstrating that the administration of testosterone propionate and estrogen benzoate in castrated rats leads to an increase in *OXTR* mRNA in the ventromedial nucleus of the hypothalamus [33,34]. Together with evidence for a role of androgens in AUD [30,61], this study’s findings corroborate an interplay between the OXTR and the androgen systems in the development and maintenance of AUD. Future studies are required to gain deeper insights into the interactions of dihydrotestosterone and testosterone with the OXTR at a molecular level and how these mechanisms differ between men and women in the context of addictive disorders.

### Strengths and Limitations

The cross-sectional (AUD versus control group), longitudinal (baseline, direct 5-day follow-up, 24-month readmission follow-up), and sex-separated design makes this study, to our knowledge, the first of its kind to assess the concentrations of OXTR in the blood of in-patients with AUD. Therefore, in this exploratory set-up, we report nominal *p* values without correction for multiple testing. By measuring alcohol-related hospital readmission, we addressed a clinically and economically relevant parameter. However, alcohol-related hospital readmissions are only a proxy for relapses. Moreover, we used a naturalistic setting, by recruiting mostly within a non-university hospital. The recruitment of a sex-balanced cohort with many female in-patients allowed us to conduct sex-separated analyses. Since the female population is highly underrepresented in AUD and neuropeptide research, we focused our attention on this often neglected but highly burdened cohort.

Limitations of the present study include, firstly, the associational study design, which does not allow for making causal inferences. Since no literature on the differences between peripheral and central OXTR concentrations is available, further research is needed to investigate how associations with blood OXTR concentrations may translate to brain function in AUD patients. Additionally, the female samples with AUD patients and healthy controls were balanced in terms of postmenopausal status because the activity of the OXT system is influenced by the menstrual cycle status [62]. Nevertheless, we had an insufficient sample size of premenopausal women to accurately account for fluctuations in the hormonal profile that occur during the menstrual cycle and are known to influence behavior [63]. The OXT system plays an important socio-sexual and socio-emotional role, and we did not account for and investigate bonding behavior, anxiety regulation, and adverse childhood experiences [6,44]. Moreover, we did not assess (epi)genetic patterns of the *OXTR* [64] as additional confounding factors. In age-mixed generally healthy adults, lower levels of *OXTR* promoter methylation and hence assumed higher *OXTR* transcription and expression were related to larger amounts of alcohol consumption in addition to reported associations with the rs53579 polymorphism [65].

Future studies should investigate the possible role that the OXTR blood concentrations may play and whether the OXTR is relevant in the development and maintenance of AUD.

## 4. Materials and Methods

### 4.1. Study Description

This study is part of the Neurobiology of Alcoholism (NOAH) project [29,31,32,52,66,67,68,69,70,71,72,73,74,75]. The participants were recruited at the psychiatric departments of the Klinikum am Europakanal Erlangen and the Universitätsklinikum Erlangen in Germany.

Our in-patient group included 200 patients who met the diagnostic criteria for AUD according to the fifth edition of the Diagnostic and Statistical Manual of Mental Disorders [76] and alcohol dependence according to the tenth revision of the International Classification of Diseases [77] after screening 988 candidates. Patients with psychiatric co-morbidities, including substance use other than alcohol or nicotine or severe somatic illness, were excluded from the study. A baseline study visit during early abstinence (24–72 h of abstinence) was conducted during which blood was drawn and behavioral parameters were assessed. Subsequently, a direct follow-up at a median of five days after the first visit took place. During the second visit, another blood sample was drawn, and a panel of psychometric testing was administered. Daily ethanol intake and lifetime consumption were determined using the Lifetime Drinking History, a structured interview based on Skinner [78]. We grouped the patients into current smokers versus non-smokers. A thorough analysis of the patients’ electronic records at both study centers for 24 months was conducted to survey alcohol-related hospital readmissions (parameters: “number of readmissions”, “days to first readmission”). For statistical analysis, days to first readmission were set to 730 days in patients without any alcohol-related readmission during the 24-month observation period. For the control group, participants were recruited via distribution of online advertisements, letters, and flyers. Following a multi-step screening procedure of 1215 subjects, 240 control subjects were included, all without any psychiatric morbidity or severe somatic illness and no psychiatric or psychotherapeutic treatment as in-patients during lifetime or as out-patients during the previous 10 years. Participants were excluded when indications of some mental illnesses were found using an adapted screening interview based on the German SCID-I (see Figure 1 in [52] for details). The German version of the 10-item Alcohol Use Disorders Identification Test (AUDIT [79]) was used to assess potential problems of alcohol consumption. Severity of tobacco dependence in patients and controls was assessed with the FTND. Non-smokers were coded with FTND scores of “0”.

### 4.2. Determination of Oxytocin Receptor, Oxytocin, and Sex Hormone Blood Concentrations, and Routine Laboratory Parameters

To minimize circadian effects, all blood samples were drawn in the morning (7:30 a.m.–11:00 a.m.). The blood vials were centrifuged for 10 min at 2000× *g*, and serum aliquots were transferred to −80 °C for storage. OXTR concentrations were quantified using the Human Oxytocin Receptor ELISA Kit from MyBioSource (MBS2506767, MyBioSource, Inc., San Diego, CA, USA) based on the sandwich enzyme-linked immunosorbent assay (ELISA) principle. Of the serum samples, 90 µL were applied in parallel to a standard curve ranging from 0.1 ng/mL to 5 ng/mL. All assays were performed in duplicates by the same operators using the same lots of reagents with a standard curve included on every 96-well plate. The coefficients of variation were 8% for intra-assay and 12% for inter-assay. The ELISA quantification methods for serum OXT, dihydrotestosterone, testosterone, estradiol, and progesterone have already been published [29,31,32,52]. From separately collected serum vials, routine markers of alcohol consumption including CDT were determined by the Central Laboratory of the Universitätsklinikum Erlangen, Germany (DIN EN ISO 15189 accredited). Blood alcohol concentrations were calculated from breath alcohol content that was determined and documented upon admission to the hospital (except for one patient who underwent a direct measurement).

### 4.3. Statistical Analyses

We used IBM SPSS for Windows 27.0 (SPSS Inc., Chicago, IL, USA) and Graph Pad Prism 5 (Graph Pad Software Inc., San Diego, CA, USA) and report medians, interquartile range (IQR), and frequencies for descriptive statistics (SPSS custom tables function). Because the OXTR concentrations deviated significantly from normal distribution according to the Kolmogorov–Smirnov test (in the total group and the sex-separated subgroups of patients and controls), non-parametric methods were employed. To compare frequencies of nominal variables and metric variables and to test for correlations, we used χ^2^, Mann–Whitney U, Wilcoxon and Spearman method, respectively. To identify the thresholds of OXTR and OXT concentrations that best separated subjects with an alcohol-related readmission from those without (including area under the curve, Youden cut-point, and related sensitivity and specificity), receiver operating characteristic curves (ROC) were computed. *p* < 0.05 for two-tailed tests was considered significant. Because of the importance of sex differences in science [80] and particularly in AUD [30,81], we analyzed men and women separately.

## 5. Conclusions

As far as we know, the present study described for the first time a difference between blood OXTR concentrations in in-patients with AUD and healthy controls. We identified several important findings: (1) higher OXTR blood concentrations in male patients with alcohol-related readmission during the 24-month follow-up than in patients without any readmission, and an association of higher baseline OXTR concentrations in male patients with more prospective alcohol related-readmissions and fewer days to the first readmission; (2) lower OXTR concentrations in smokers versus non-smokers in the female patient and control groups; (3) a correlation of lower baseline OXTR concentrations with higher age in male and female healthy control subjects; and (4) correlations between OXTR concentrations and levels of androgens in both male and female healthy controls. These results provide novel insights into the role of OXTR in AUD. Future studies are necessary to build on the available knowledge of the possible clinical implications of the peripheral blood OXTR in addiction research.

## Figures and Tables

**Table 1:** Demographic characteristics of the male and female groups of in-patients with AUD and healthy controls.

	AUD Group				Control Group				AUD Group vs. Control Group	
	N	M/F	IQR		N	M/F	IQR		U or χ^2^	*p*
**Men**										
Age (years)	113	48	40	53	133	48	38	56	7369	0.794 ^#^
Fasting (%)	103	16			127	24			2.8	0.097 ^+^
Alcohol concentration at admission (‰)	108	1.7	0.5	2.4		-				
Number of previous withdrawal treatments	89	6	2	12		-				
CDT (nephelometry, %)	113	2.8	1.9	4.0	132	1.5	1.3	1.7	1636	**<0.001** ^#^
AUDIT score		-			125	4	3	6		
Smokers (%)	104	78			133	22			73.8	**<0.001** ^+^
FTND score	99	5.0	3.0	7.0	130	0.0	0.0	3.0	2556	**<0.001** ^#^
24-month alcohol-related readmissions										
Risk	113	0.67								
Total number	113	2	0	4		-				
Latency (days)	113	285	57	≥730		-				
**Women**										
Age (years)	87	48	42	55	107	49	39	55	4542	0.772 ^#^
Fasting (%)	80	18			101	26			1.8	0.184 ^+^
Postmenopausal status (%)	73	51			100	44			0.8	0.384 ^+^
Alcohol concentration at admission (‰)	85	1.2	0.1	1.8		-				
Number of previous withdrawal treatments	58	5	2	11		-				
CDT (nephelometry, %)	87	1.9	1.6	2.5	107	1.5	1.3	1.6	1415	**<0.001** ^#^
AUDIT score		-			96	3	2	4		
Smokers (%)	78	77			107	19			62.3	**<0.001** ^+^
FTND score	75	5.0	0.5	7.0	103	0.0	0.0	2.0	1757	**<0.001** ^#^
24-month alcohol-related readmissions										
Risk	87	0.53								
Total number	87	1	0	3		-				
Latency (days)	87	625	90	≥730		-				

The table shows the valid number of subjects analyzed (N), medians (M) or relative frequencies (F), interquartile ranges (IQR), and the results of ^#^ Mann-Whitney U and ^+^ χ^2^ tests. AUD, alcohol use disorder; AUDIT, Alcohol Use Disorders Identification Test; CDT, carbohydrate-deficient transferrin; FTND, Fagerström Test for Nicotine Dependence. *p* < 0.05 in bold.

**Table 2:** Cross-sectional and longitudinal comparison of oxytocin receptor blood concentrations between male and female in-patients with alcohol use disorder and healthy control subjects.

	AUD Group				Control Group				AUD vs. Control Group		T0 vs. T1	
	N	M	IQR		N	M	IQR		U	*p* ^#^	z	*p* ^§^
Men												
OXTR T0	113	0.417	0.273	0.642	133	0.437	0.288	0.678	7351	0.769	−0.62	0.533
OXTR T1	94	0.479	0.261	0.635					6239	0.980		
Women												
OXTR T0	87	0.470	0.317	0.749	107	0.428	0.230	0.758	4175	0.218	−1.37	0.172
OXTR T1	69	0.465	0.281	0.664					3469	0.500		

The table shows medians (M) and interquartile ranges (IQR) and results of ^#^ Mann–Whitney U and ^§^ Wilcoxon-tests. AUD, alcohol use disorder; OXTR, oxytocin receptor (ng/mL); T0, baseline during early abstinence; T1, direct follow-up at median 5 days following T0.

## Data Availability

Data are available upon request.

## Supplementary Materials

The following supporting information can be downloaded at: https://www.mdpi.com/article/10.3390/ijms23179940/s1.
