# Supplementary material for: Elevated Oxytocin Receptor Blood Concentrations Predict Higher Risk for, More, and Earlier 24-Month Hospital Readmissions after In-Patient Detoxification in Males with Alcohol Use Disorder
Source: Int J Mol Sci. 2022 Sep 1;23(17):9940. doi: 10.3390/ijms23179940 (PMC9455990; doi:10.3390/ijms23179940)
Supplement: Supplementary file 1 [file ijms-23-09940-s001.zip › ijms-1879730-supplementary.pdf]

Article

Elevated Oxytocin Receptor Blood Concentrations Predict Higher Risk for, More, and Earlier 24-Month Hospital Readmissions after In-Patient Detoxification in Males with Alcohol Use Disorder

Christiane Mühle, Massimiliano Mazza, Christian Weinland, Claudia von Zimmermann, Patrick Bach, Falk Kiefer, Valery Grinevich, Iulia Zoicas, Johannes Kornhuber, Bernd Lenz

Supplementary Material

Supplementary Table S1. Risk of alcohol-related readmission during the 24-month follow-up period depending on oxytocin and oxytocin receptor serum levels using male-specific Youden cut-off points of 37.6 pg/mL for oxytocin (based on data from [52], resulting in a sensitivity of 0.42, a specificity of 0.78, area under the curve 0.611, standard error under the non-parametric assumption 0.056, $p = 0.046$) and of 0.351 ng/mL for the oxytocin receptor (see text for details; the groups differed significantly in terms of readmission risk, $\chi^2 = 12.6$, $p = 0.006$)

Number (percentage) of male patients with alcohol-related hospital re-admission	OXTR < 0.351 ng/mL	OXTR ≥ 0.351 ng/mL
OXT < 37.6 pg/mL	19 out of 37 (51.4%)	25 out of 36 (69.4%)
OXT ≥ 37.6 pg/mL	3 out of 7 (42.9%)	29 out of 33 (87.9%)

OXT, oxytocin; OXTR oxytocin receptor.

Supplementary Table S2. Comparison of baseline oxytocin receptor serum levels (ng/mL) in male and female in-patients with alcohol use disorder between those with and those without prospective 24-month hospital readmission

	With readmission				Without readmission					
	N	M	IQR		N	M	IQR		U	*p*
Male in-patients with AUD	76	0.456	0.317	0.712	37	0.319	0.185	0.500	939	**0.004**
Female in-patients with AUD	46	0.491	0.288	0.724	41	0.452	0.328	0.749	913	0.799

AUD, alcohol use disorder; the table shows medians (M), interquartile ranges (IQR), and results from Mann-Whitney U tests. $p < 0.05$ in bold.

Supplementary Table S3. Spearman correlations between baseline oxytocin receptor serum levels, number of alcohol-related readmissions, and latency to first alcohol-related readmission during the prospective 24-month period

		Alcohol-related hospital readmission			
		Number		Latency (days)	
	N	ρ	*p*	ρ	*p*
Male in-patients with AUD	113	0.249	**0.008**	–0.268	**0.004**
Female in-patients with AUD	87	–0.021	0.848	0.000	0.997

AUD, alcohol use disorder; $p < 0.05$ in bold.

Supplementary Table S4. Baseline oxytocin receptor serum levels (ng/mL) and smoking status in male and female groups of in-patients with alcohol use disorder and healthy control subjects.

	Smoker				Non-smoker					
	N	M	IQR		N	M	IQR		U	$p^{\#}$
Male in-patients with AUD	81	0.412	0.246	0.608	23	0.442	0.302	0.903	745	0.144
Female in-patients with AUD	60	0.411	0.252	0.610	18	0.668	0.470	1.301	267	**0.001**
Male controls	29	0.402	0.304	0.653	104	0.438	0.282	0.700	1380	0.486
Female controls	20	0.253	0.175	0.429	87	0.494	0.250	0.894	503	**0.003**

AUD, alcohol use disorder; medians (M), interquartile ranges (IQR). #Mann-Whitney U tests. $p < 0.05$ in bold.

Supplementary Table S5. Spearman correlations between baseline oxytocin receptor serum levels and age in male and female groups of in-patients with alcohol use disorder and healthy control subjects.

		Age	
	N	ϱ	p
Male in-patients with AUD	113	0.026	0.784
Female in-patients with AUD	87	0.039	0.721
Male controls	133	–0.192	**0.027**
Female controls	107	–0.246	**0.011**

AUD, alcohol use disorder. $p < 0.05$ in bold.

Supplementary Table S6. Spearman correlations between baseline oxytocin receptor serum levels, dihydrotestosterone, testosterone, estradiol, and progesterone in male and female groups of in-patients with alcohol use disorder and healthy control subjects

		Dihydro-testosterone		Testosterone		Estradiol		Progesterone	
	N	ϱ	p	ϱ	p	ϱ	p	ϱ	p
Male in-patients with AUD	113	0.073	0.445	–0.005	0.956	–0.004	0.964	–0.016	0.864
Female in-patients with AUD	87	0.025	0.819	0.000	1.000	0.119	0.272	–0.010	0.926
Male controls	133	0.189	**0.030**	0.134	0.124	0.057	0.516	0.165	0.057
Female controls	107	0.148	0.129	0.281	**0.003**	0.136	0.163	0.186	0.055

AUD, alcohol use disorder. $p < 0.05$ in bold.
